# Atrophy related neuroimaging biomarkers for neurological and cognitive function in Wilson disease

**DOI:** 10.1186/s42466-025-00401-3

**Published:** 2025-07-01

**Authors:** Ann Carolin Hausmann, Christian Rubbert, Silja K. Querbach, Vivien Lorena Ivan, Alfons Schnitzler, Christian Johannes Hartmann, Julian Caspers

**Affiliations:** 1 Institute of Clinical Neuroscience and Medical Psychology, Medical Faculty and University Hospital Düsseldorf, Heinrich-Heine-University Düsseldorf, Düsseldorf, Germany; 2 Department of Diagnostic and Interventional Radiology, Medical Faculty and University Hospital Düsseldorf, Heinrich-Heine-University Düsseldorf, Düsseldorf, Germany; 3 Department of Neurology, Medical Faculty and University Hospital Düsseldorf, Heinrich-Heine-University Düsseldorf, Düsseldorf, Germany

**Keywords:** Wilson disease, Magnetic resonance imaging, Atrophy, *BrainAGE*, Cognition

## Abstract

**Background:**

Although brain atrophy is a prevalent finding in Wilson disease (WD), its role as a contributing factor to clinical symptoms, especially cognitive decline, remains unclear. The objective of this study was to investigate different neuroimaging biomarkers related to grey matter atrophy and their relationship with neurological and cognitive impairment in WD.

**Methods:**

In this study, 30 WD patients and 30 age- and sex-matched healthy controls were enrolled prospectively and underwent structural magnetic resonance imaging (MRI). Regional atrophy was evaluated using established linear radiological measurements and the automated workflow* v*olumetric *e*stimation of *g*ross *a*trophy a*n*d *b*rain *age l*ongitudinally (*veganbagel*) for age- and sex-specific estimations of regional brain volume changes. Brain Age Gap Estimate (*BrainAGE*), defined as the discrepancy between machine learning predicted brain age from structural MRI and chronological age, was assessed using an established model. Atrophy markers and clinical scores were compared between 19 WD patients with a neurological phenotype (neuro-WD), 11 WD patients with a hepatic phenotype (hep-WD), and a healthy control group using Welch’s ANOVA or Kruskal–Wallis test. Correlations between atrophy markers and neurological and neuropsychological scores were investigated using Spearman’s correlation coefficients.

**Results:**

Patients with neuro-WD demonstrated increased third ventricle width and bicaudate index, along with significant striatal-thalamic atrophy patterns that correlated with global cognitive function, mental processing speed, and verbal memory. Median *BrainAGE* was significantly higher in patients with neuro-WD (8.97 years, interquartile range [IQR] = 5.62–15.73) compared to those with hep-WD (4.72 years, IQR = 0.00–5.48) and healthy controls (0.46 years, IQR = − 4.11–4.24). Striatal-thalamic atrophy and *BrainAGE* were significantly correlated with neurological symptom severity.

**Conclusions:**

Our findings indicate advanced predicted brain age and substantial striatal-thalamic atrophy patterns in patients with neuro-WD, which serve as promising neuroimaging biomarkers for neurological and cognitive functions in treated, chronic WD.

**Supplementary Information:**

The online version contains supplementary material available at 10.1186/s42466-025-00401-3.

## Introduction

Wilson disease (WD) is an autosomal recessive disorder of copper metabolism that leads to liver disease, neurological dysfunction, or neuropsychiatric symptoms [1]. In addition, cognitive impairment may manifest in multiple domains, including attention, memory, executive function, and processing speed [2–5]. Patients with a neurological phenotype demonstrate a diminished health-related quality of life, with cognitive decline representing a major contributing factor [6]. However, only few studies have investigated the neuroanatomical basis of cognitive deficits in WD.

Brain atrophy is a prevalent neuroimaging feature in WD, with the most pronounced volume loss in deep grey matter (GM) nuclei, particularly the caudate nucleus, putamen, globus pallidus, and thalamus [7–11]. As atrophy persists despite decoppering treatment, it is put forth as a useful biomarker for chronic and irreversible brain damage [9, 12]. While linear radiological measurements serve as surrogate markers of atrophy, they may lack the sophistication needed for precise assessment, and volumetric analyses within a clinical context remain constrained by the requisite time, expertise, and reference data. To address these challenges, automated workflows offer the prospect of observer-independent, individual-level quantification of volumetric brain changes relative to large normative cohorts. Automated atrophy estimation tools have demonstrated the potential to facilitate the investigation of disease-specific regional atrophy patterns [13]. Furthermore, the Brain Age Gap Estimate (*BrainAGE*), defined as the discrepancy between an individual’s machine learning predicted brain age from structural magnetic resonance imaging (MRI) and their chronological age, has emerged as an indicator of brain health [14, 15]. Increased *BrainAGE*, or accelerated “brain aging”, has been documented in several neurodegenerative diseases, e.g., Alzheimer’s and Parkinson’s disease [16, 17]. The value of these novel markers in WD remains to be evaluated.

Deep GM atrophy has been proposed as a promising correlate of neurological impairment in WD [8, 11, 18, 19]. Moreover, previous studies have revealed correlations between basal ganglia atrophy and executive dysfunction [20], and between cortical thickness of the right orbitofrontal gyrus and prospective memory in WD patients [21]. Nevertheless, given the inconsistent evidence [22, 23], the clinical relevance of atrophy for WD-related neurological and cognitive symptoms remains a subject of ongoing debate.

The objective of this study was to investigate different neuroimaging biomarkers related to atrophy in WD and their association with clinical symptoms. For this, conventional atrophy measurements, automated atrophy estimation as well as automated brain age prediction are assessed from structural MRI and evaluated regarding their correlations with neurological and neuropsychological scores in 30 WD patients and 30 age- and sex-matched healthy controls.

## Methods

### Study participants

In this prospective, cross-sectional study, 36 WD patients were recruited from the WD outpatient clinic of the Department of Neurology of the University Hospital Düsseldorf between March 2022 and January 2024. Additionally, 30 age- and sex-matched healthy controls were recruited. Patients aged ≥ 18 years with an established diagnosis of WD in accordance with the Leipzig criteria were included [24]. If an initial Leipzig score was not available, it was determined based on a comprehensive review of medical records. Exclusion criteria comprised contraindications to MRI and a history of an unrelated neurological disease of different aetiology [25, 26] or other severe medical condition that would interfere with the study assessments. Patients were classified as having a neurological phenotype (neuro-WD) if they initially had or developed significant neurological impairment during the course of the disease. Those with no initial or history of significant neurological impairment were classified as having a hepatic phenotype (hep-WD).

### Clinical assessments

Patients underwent a neurological examination by an expert neurologist in the field of movement disorders (CJH, 15 years of experience) and were scored on the Unified WD Rating Scale neurological subscale (UWDRS-N). The Mini-Mental State Examination (MMSE) was performed to assess global cognitive functioning. To mitigate potential confounding effects of WD-related motor impairment that could impede handwriting ability, the oral version of the Symbol Digit Modalities Test (SDMT) was administered to evaluate sustained attention and mental processing speed. Verbal working memory was tested using the Rivermead Behavioural Memory Test-story subtest (RBMT-S). The performance in the interference trial of the Colour-Word Interference Test (CW-INT) was noted to evaluate executive functions. *Z*-transformation was employed for SDMT, RBMT-S, and CW-INT scores using normative data.

### Imaging acquisition

High-resolution structural MRI was acquired on a 3 T Siemens Prisma scanner (Siemens Healthineers, Erlangen, Germany). T_1_-weighted images were obtained using acquisition parameters adapted from the Lifespan Human Connectome Project in Aging [27] (Additional file 1). The acquired scans were visually inspected to identify significant motion artifacts and were repeated when necessary.

### Common radiological measurements

Third ventricle width (TVW) was measured as an indicator of central atrophy, consistent with the chronic damage—atrophy subscore of the WD MRI severity scale [28]. As previously studied in the context of WD [29], the bicaudate index (BI), defined as the minimum distance between the caudate nuclei’s heads divided by the transverse diameter of brain tissue at that level, was employed as a marker of caudate atrophy [30]. Measurements were performed by two independent raters (ACH; VLI) in axial T_1_-weighted images using the local Sectra IDS7 PACS (v25.2; Sectra AB, Linköping, Sweden; Additional file 1).

### Automated atrophy estimation

We employed the *v*olumetric *e*stimation of *g*ross *a*trophy a*n*d *b*rain *age l*ongitudinally (*veganbagel*) workflow for automated regional brain atrophy estimation [13]. In brief, *veganbagel* automatically preprocesses three-dimensional T_1_-weighted images in a standardized manner (i.e., GM segmentation, normalization, modulation, and smoothing) and employs a voxel-wise comparison of individual GM maps with the mean and standard deviation of corresponding normative templates to generate *z*-score maps, or “atrophy maps,” which reflect age- and sex-specific estimations of GM volume deviations [13]. The current study used precomputed templates for each combination of age (including actual age ± 2 years) and sex, derived from 1004 subjects from the enhanced Nathan Kline Institute - Rockland Sample (eNKI [31]). The resulting *z*-maps are transformed back into subject space, color-coded, and fused with the original structural images [13]. Voxels demonstrating a decrease exceeding the 2.5 standard deviations cutoff point relative to their respective mean (blue color-coding) are considered atrophic. 

**Fig. 1:**
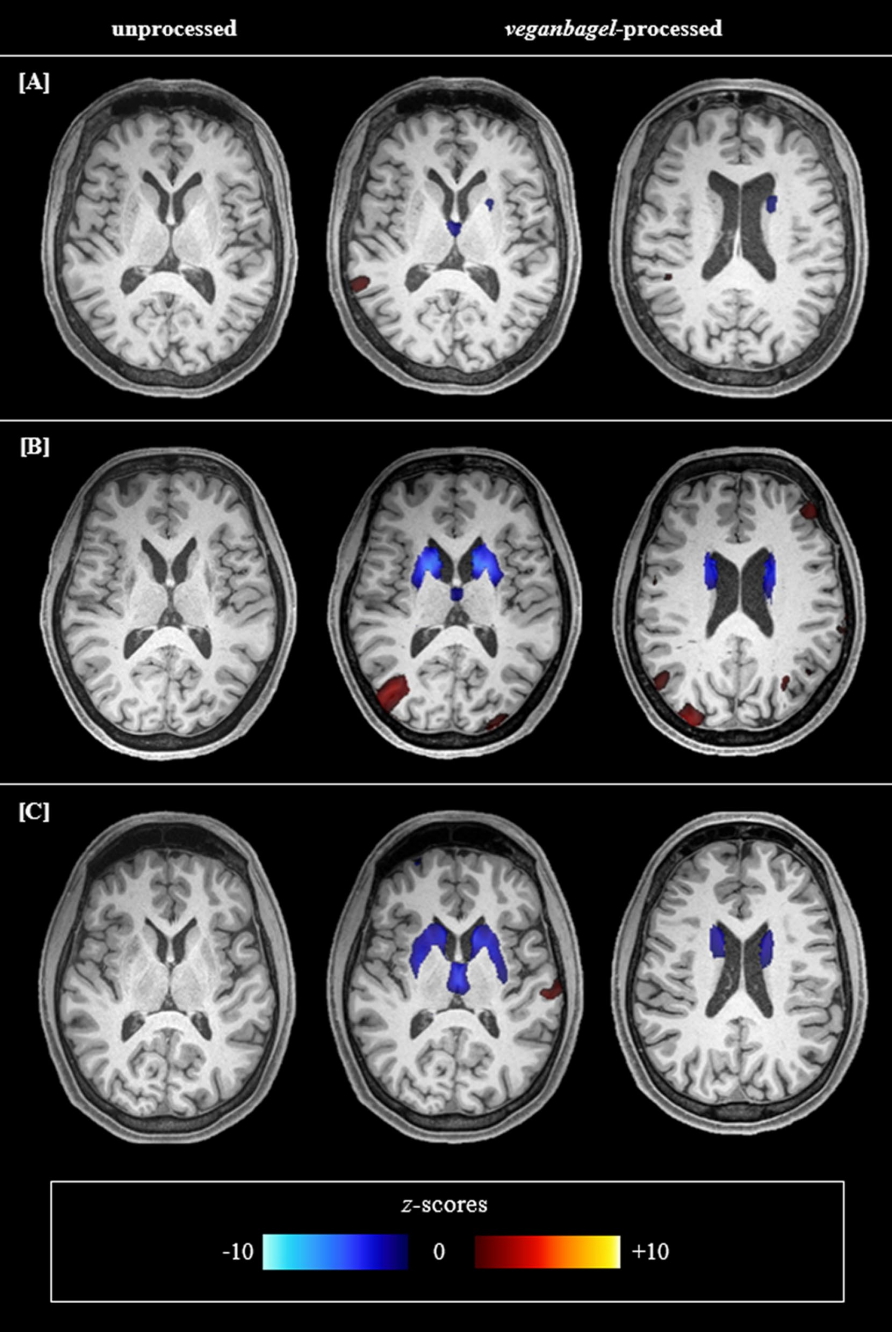
Striatal-thalamic atrophy patterns in patients with Wilson disease. As an illustration of the striatal-thalamic atrophy patterns observed in most atrophy maps of patients with neurological Wilson disease, the native T_1_-weighted image and two corresponding representative slices of *veganbagel*-generated atrophy maps of three patients are presented. For instance, **A** shows a patient with comparatively mild striatal-thalamic atrophy, **B** shows a patient with severe striatal atrophy, and **C** shows a patient with marked striatal atrophy and additional thalamic atrophy. Color-coding indicates deviations of grey matter volumes compared to respective age- and sex-specific normative templates (blue indicating *z*-scores < − 2.5, red indicating *z*-scores > 2.5). Visual assessment indicated that atrophy was most pronounced in the putamen, and that the mediodorsal and anterior nuclei appeared to be the predominant regions of thalamic volume loss

In this study, images were analysed via an Docker-instance of *veganbagel* (commit 8e022a1), which implements the standalone version of the Computational Anatomy Toolbox (vCAT12.8.1 [32]) for Statistical Parametric Mapping (SPM12, v7771 [33]). A thorough visual inspection of atrophy maps was conducted to identify patterns of WD-related atrophy. Consistent with prior research, the dorsal striatum (i.e., putamen and caudate nucleus) and thalamus were identified as key regions of interest (Fig. 1). To facilitate group-level comparisons, a visual rating scale for striatal-thalamic atrophy was defined (Table 1) and atrophy maps were scored by two independent raters (ACH; VLI).

**Table 1:** Visual rating scale for striatal-thalamic atrophy in Wilson disease

Example	Score	Grade of atrophy	Definition
**Striatal-thalamic atrophy score**			
**Striatum**			
	0	absent	no atrophied voxels within the striatum
	1	minimal/mild	isolated or sporadically distributed atrophied voxels within the putamen or caudate nucleus
	2	moderate	marked clusters of atrophied voxels within the putamen and/or caudate nucleus
	3	severe	extensive clusters of atrophied voxels across the putamen and caudate nucleus
**Thalamus**			
	0	absent	no atrophied voxels within the thalamus
	1	minimal/mild	isolated or sporadically distributed atrophied voxels within the thalamus
	2	moderate	marked clusters of atrophied voxels within one or more thalamic nuclei
	3	severe	extensive clusters of atrophied voxels throughout several thalamic nuclei

In accordance with a previous study on Alzheimer’s disease patients [13], the age- and sex-specific atrophy maps generated by *veganbagel* were subjected to a visual evaluation in order to facilitate inter-subject atrophy evaluation and to enhance the approach’s feasibility. Therefore, a visual rating scale was defined, and each individual atrophy map was scored for striatal and thalamic atrophy, with a score ranging from 0 to 3 for each. These scores are then summed to calculate the striatal-thalamic atrophy score, ranging from 0 to 6.

### Brain age prediction

Brain age was predicted using the validated, best-performing workflow from a previous evaluation of various workflows and machine learning algorithms for automated brain age estimation [16], which were integrated in *veganbagel*. This workflow includes the standardized preprocessing of T_1_-weighted images with CAT12 as described above, followed by resampling voxels to a spatial resolution of 4 mm and a dimensionality reduction via principal component analysis. Subsequently, the brain age of an individual is predicted from their GM image via a Gaussian process regression trained to predict age from GM features on 2953 healthy subjects from several large-scale neuroimaging cohorts, including 1000BRAINS [34], the eNKI [31], the Cambridge Centre for Ageing and Neuroscience [35], and Information eXtraction from Images [36] [16]. In this study, T_1_-weighted images were subjected to these automated processing steps to predict brain age in our sample. *BrainAGE* was calculated by subtracting actual age from predicted age, with scores > 0 indicating accelerated brain aging [15, 16].

### Statistical analysis

Normality was evaluated using Shapiro–Wilk tests. To assess inter-rater reliability, intraclass correlation coefficients (ICC) or Cohen’s kappa were calculated. Group differences in demographics were evaluated using Fisher-Freeman-Halton exact tests or analysis of variance (ANOVA). Atrophy markers and clinical scores were compared among the three groups (neuro-WD, hep-WD, and controls) using Welch’s ANOVAs or Kruskal–Wallis tests, followed by planned contrast analyses or Dunn’s tests. Within-group comparisons of predicted and actual age were conducted using paired *t*-tests. In the total sample of 30 WD patients, the relationship between and within atrophy markers and clinical scores was investigated using Spearman’s correlation coefficients. Statistical analyses were conducted using SPSS (v29; IBM Corp, New York) with *p* ≤ 0.05 considered significant, and the Bonferroni-Holm correction was applied for multiple comparisons. Figures were created using R (v4.4.1).

## Results

### Demographic and clinical characteristics

Of the 36 enrolled patients, five did not complete the MRI acquisition due to technical issues or discomfort and one was excluded due to an incidental finding. Thus, 30 patients, 19 with neuro-WD and 11 with hep-WD, and 30 controls were included in subsequent analyses (Table 2). None of the participants exhibited signs of hepatic encephalopathy or had undergone liver transplantation.

**Table 2:** Demographics and clinical characteristics

	Neuro-WD (n = 19)	Hep-WD (n = 11)	Controls (n = 30)	*p*
**Demographics**				
Male/female, n	5/14	3/8	8/22	>0.999
Age, years	43.5 (± 11.4)	34.3 (± 12.3)	40.1 (± 12.3)	0.143
Education, years	15.6 (± 2.5)	15.2 (± 2.7)	14.8 (± 1.9)	0.495
Disease duration, years	24.2 (± 11.0)	17.5 (± 9.4)	–	.0102
Treatment (d-penicillamine/trientine/chelator + zinc)	8/10/1	4/5/2	–	0.733
**Neurological scores**				
UWDRS-N	6 [4–15]	2 [0–4]	0 [0–0]	<0.001
Neurological symptoms, %				
Parkinsonism	68%			
Tremor	53%			
Ataxia	42%			
Dysarthria	32%			
Impaired handwriting	32%			
Dystonia	26%			
Rigidity	26%			
Chorea	5%			
**Neuropsychological scores**				
MMSE	29 [28–29]	30 [29–30]	30 [29–30]	0.006
SDMT, *z*	− 0.55 (± 0.85)	0.10 (± 0.87)	0.55 (± 0.79)	<0.001
RBMT-S, *z*	− 0.35 (± 1.10)	0.44 (± 1.31)	0.57 (± 0.85)	0.011
CW-INT, *z*	0.14 (± 0.67)	0.61 (± 0.63)	0.66 (± 0.64)	0.023

Data is presented as counts (%), mean (± standard deviation), or median [interquartile range]

Neuro-WD, neurological Wilson disease; Hep-WD, hepatic Wilson disease; UWDRS-N, Unified Wilson’s Disease Rating Scale-neurological subscale; MMSE, Mini-Mental State Examination; SDMT, Symbol Digit Modalities Test; RBMT-S, Rivermead Behavioural Memory Test-story subtest; CW-INT, Color-Word Interference Test-interference trial

UWDRS-N scores were higher in the neuro-WD group relative to the hep-WD (*p* = 0.003) and control group (*p* < 0.001). Moreover, neuro-WD patients performed worse on the SDMT and RBMT-S compared to hep-WD patients (*p* = 0.043; *p* = 0.047) and controls (*p* < 0.001; *p* = 0.004), and scored lower on the MMSE (*p* = 0.005) and CW-INT (*p* = 0.008) relative to controls. Clinical symptoms did not differ between hep-WD patients and controls. In WD patients, a correlation was observed between UWDRS-N and SDMT scores (*r*_*s*_ = − 0.52, *p* = 0.007).

### Common radiological measurements

The inter-rater reliability was excellent regarding TVW and minimal intercaudate distance (ICC = 0.98 and 0.93), and good regarding brain tissue diameter at the caudate level (ICC = 0.83), all *p* < 0.001. Given the high level of agreement, measurements of both raters were averaged, and the BI was calculated.

Patients with neuro-WD demonstrated greater TVW and BI relative to hep-WD patients and controls, all *p* < 0.001 (Table 3). Measurements did not differ between hep-WD patients and controls (*p* = 0.643; *p* = 0.113).

**Table 3:** Group differences in regional atrophy assessments

	Neuro-WD	Hep-WD	Controls	*p*
**Common radiological measurements**				
Third ventricle width	5.97 (± 2.50)	2.68 (± 1.90)	2.39 (± 1.14)	<.001*
Bicaudate index	0.13 (± 0.03)	0.08 (± 0.02)	0.09 (± 0.02)	<.001*
**Atrophy map ratings**				
Striatal-thalamic atrophy	3 [1–5]	0 [0–0]	0 [0–0]	<.001*
Striatal atrophy	2 [0–3]	0 [0–0]	0 [0–0]	<.001*
Thalamic atrophy	1 [0–2]	0 [0–0]	0 [0–0]	<.001*

The mean (± standard deviation) of common radiological measurements of atrophy is shown in millimeters. For atrophy map ratings, the median [interquartile range] is indicated. Welch’s ANOVA or Kruskal–Wallis tests revealed significant group differences in all parameters

Neuro-WD, neurological Wilson disease; Hep-WD, hepatic Wilson disease

*p-value <.001

### Automated atrophy estimation

There was excellent inter-rater reliability for atrophy map ratings of the striatum (*κ* = 0.87) and thalamus (*κ* = 0.84), all *p* < 0.001. Discrepancies were resolved through consensus, and striatal-thalamic atrophy scores were calculated.

Automated atrophy estimation revealed striatal-thalamic atrophy patterns in the majority of neuro-WD patients (Fig. 1). In the neuro-WD group, striatal atrophy was identified in 14 patients (74%; three with mild, five with moderate, six with severe atrophy), while thalamic atrophy was detected in 12 patients (63%; four with mild, six with moderate, two with severe atrophy). In the control group, mild atrophy was identified in the striatum of five (17%) subjects and in the thalamus of two (7%) subjects. None of the hep-WD patients demonstrated striatal-thalamic atrophy. Striatal-thalamic atrophy scores were significantly higher in neuro-WD than in hep-WD patients and controls, all *p* < 0.001 (Table 3). There was no significant difference between the hep-WD and the control group (*p* = 0.361).

### Brain age prediction

In healthy controls, age was well predicted by the implemented model (*r* = 0.91, mean absolute error [MAE] = 4.16). The estimated age (*M* = 40.31 ± 12.33) did not differ from chronological age (*M* = 40.12 ± 11.49; *p* = 0.418) in controls, yielding a median *BrainAGE* of 0.46 years. Within both patient groups, predicted age was significantly higher than chronological age (neuro-WD: *M* = 55.36 ± 13.25 vs. 43.48 ± 11.38, *p* < 0.001; hep-WD: *M* = 38.42 ± 12.54 vs. 34.34 ± 12.27, *p* = 0.019), resulting in a median *BrainAGE* of 8.97 years in neuro-WD and 4.72 years in hep-WD.

**Fig. 2:**
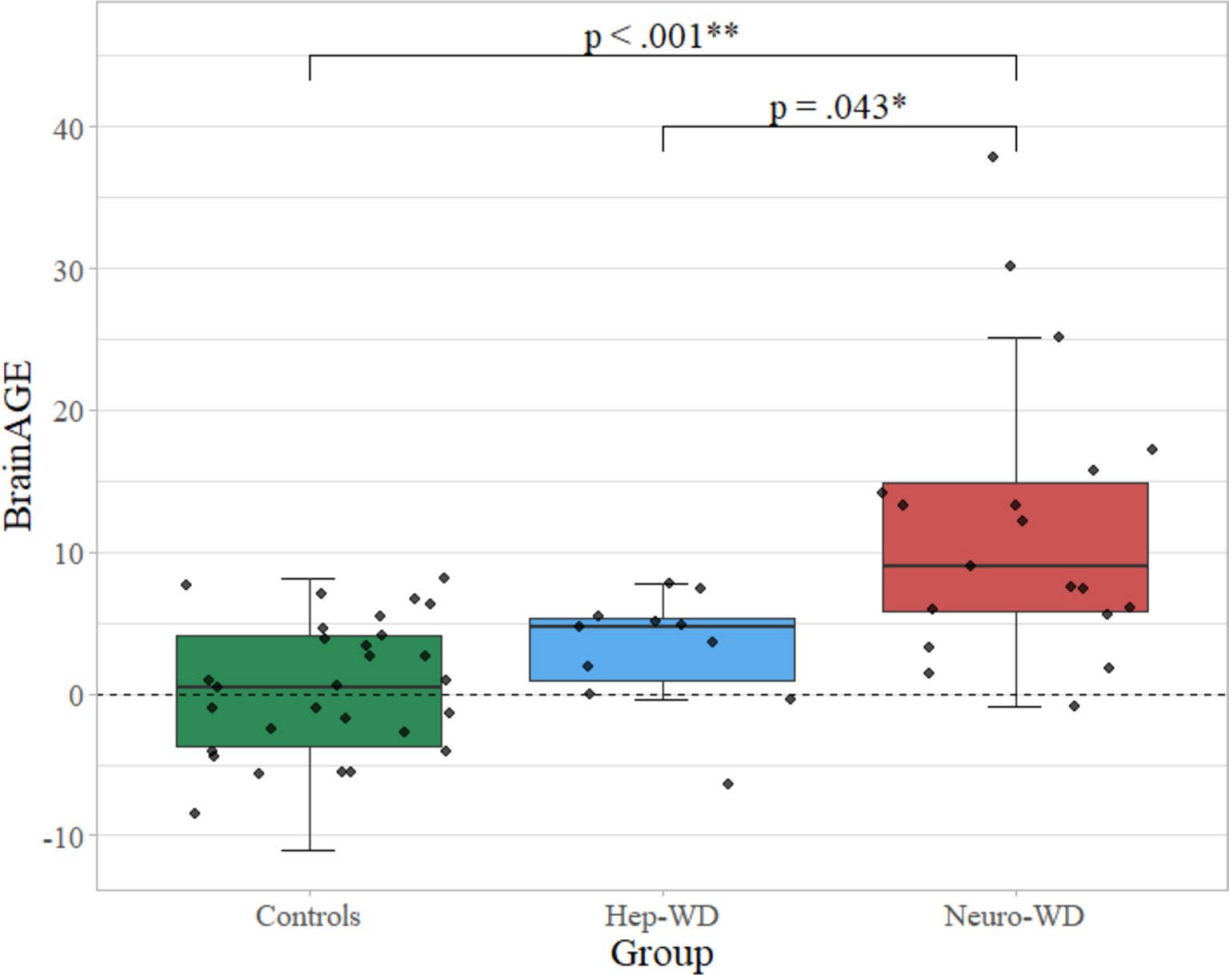
*BrainAGE* of patients with Wilson disease and controls. Medians and interquartile ranges of *BrainAGE* scores in the control group, hep-WD group, and neuro-WD group are shown. A Kruskal–Wallis test revealed that *BrainAGE* significantly differed among the three groups (*p* <.001). Significant post-hoc comparisons are indicated. *BrainAGE* = Brain Age Gap Estimate; Hep-WD = hepatic Wilson disease; Neuro-WD = neurological Wilson disease; * *p*-value <0.050; ** *p*-value <0.001

Neuro-WD patients demonstrated significantly higher *BrainAGE* than hep-WD patients and controls (Fig. 2). *BrainAGE* was not significantly different between hep-WD patients and controls (*p* = 0.161).

### Clinical correlations

**Fig. 3:**
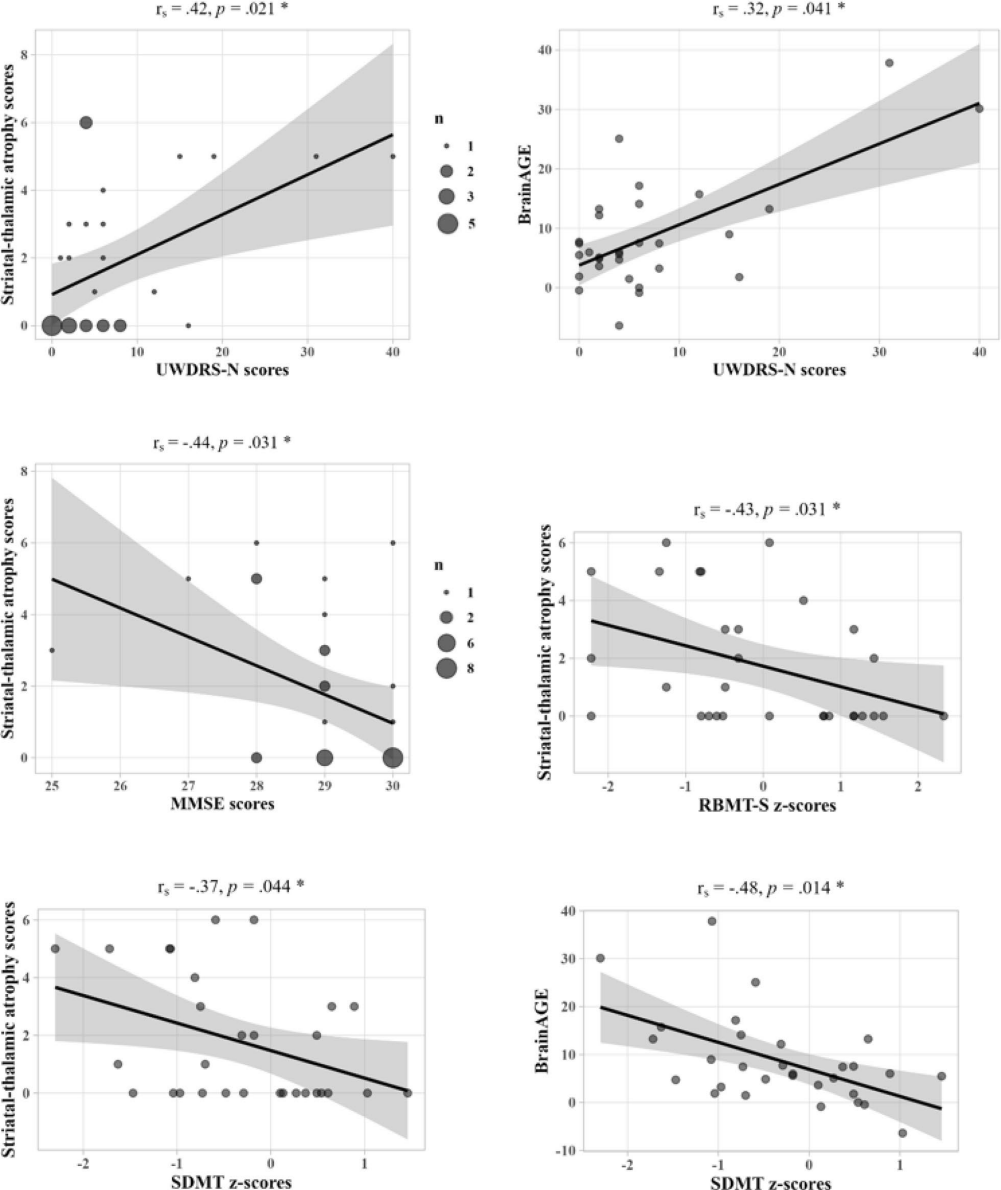
Clinical correlations of striatal-thalamic atrophy and *BrainAGE*. Significant correlations between clinical scores, striatal-thalamic atrophy scores, and *BrainAGE* (in years) in the total sample of WD patients are shown (Bonferroni-Holm corrected* p* ≤0.05). Striatal-thalamic atrophy scores demonstrated a significant correlation with neurological scores and the majority of cognitive scores, including MMSE, RBMT-S, and SDMT. *BrainAGE* was also significantly correlated with neurological scores; however, except for SDMT scores, the associations between *BrainAGE* and neuropsychological scores lost statistical significance following multiple comparison correction. A comparison of the variance explained in UWDRS-N scores by the novel neuroimaging biomarkers in addition to common radiological measurements can be found in Additional File 1. UWDRS-N = Unified Wilson’s Disease Rating Scale-neurological subscale; *BrainAGE* = Brain Age Gap Estimate; MMSE = Mini-Mental State Examination; SDMT = Symbol Digit Modalities Test; RBMT-S = Rivermead Behavioural Memory Test-story subtest; WD = Wilson disease; r_s_ = Spearman’s correlation coefficients; * *p*-value <0.050

Striatal-thalamic atrophy scores were strongly positively correlated with TVW (*r*_*s*_ = 0.58), BI (*r*_*s*_ = 0.69), and *BrainAGE* (*r*_*s*_ = 0.74), all *p* < 0.001. *BrainAGE* also correlated positively with TVW (*r*_*s*_ = 0.61) and BI (*r*_*s*_ = 0.58), both *p* < 0.001. Additionally, striatal-thalamic atrophy scores and *BrainAGE* were significantly correlated with several clinical scores (see Fig. 3). Correlations between clinical scores, TVW, and BI are shown in Additional file 1. There were no correlations between atrophy markers and age or disease duration.

## Discussion

This study systematically investigated different neuroimaging biomarkers related to atrophy and their correlations with clinical symptoms in WD. Our findings offer compelling evidence for a consistent pattern of striatal-thalamic atrophy in most patients with neuro-WD, which was identified as a correlate of neurological symptoms, global cognitive function, mental processing speed, and verbal memory. We further present the first evidence indicating that neuro-WD leads to an estimated 9-year increase in predicted brain age, which correlates with neurological impairment. Thus, this study provides implications for the clinical relevance of GM atrophy and emphasizes the potential of disease-related atrophy patterns and *BrainAGE* as valuable neuroimaging biomarkers in chronic, treated WD.

Recent advancements in neuroimaging analysis hold considerable promise for enhancing the clinical feasibility of volumetric analyses. However, their application in WD has not yet been investigated. To address this research gap, an automated workflow for regional atrophy estimation was employed, revealing striatal-thalamic atrophy patterns in the majority of neuro-WD patients. This finding aligns with volumetric studies that identified substantial volume loss in the putamen, caudate nucleus, and thalamus compared to healthy controls [8, 11, 22, 37]. Combined with the strong correlations with TVW and BI, which are recognized indicators of central and caudate atrophy [28, 30], this lends support to the validity and feasibility of automated atrophy estimation as implemented in *veganbagel* for detecting individual-level atrophy in the striatum and thalamus in WD.

Furthermore, we demonstrated that brain aging in neuro-WD patients was significantly advanced by approximately 9 years, which exceeds the observed progression in Alzheimer’s patients (~ 4.5–7 years) when utilizing the same prediction model [16]. This places the *BrainAGE* of neuro-WD within the upper range when compared to other neurodegenerative or psychiatric diseases, including schizophrenia (~ 2.5 years, [38]) Parkinson’s disease (~ 3–4 years, [17, 39]), mild cognitive impairment (~ 3–8 years, [40]), or multiple sclerosis (~ 10 years, [39]). Given the largely irreversible nature of copper-induced atrophy and the potential for progression even under decoppering treatment [9, 12], significant GM atrophy and, thus, a highly positive *BrainAGE* was anticipated in a chronically ill sample such as ours. Considering the strong correlation with striatal-thalamic atrophy scores, the observed increase in *BrainAGE* may be highly driven by regional changes in deep GM. The validity of our findings is substantiated by longitudinal data, which indicates increased annualized atrophy rates of approximately 5% in neuro-WD compared to hep-WD patients [41].

It is noteworthy that we found brain aging to be advanced by approximately 5 years in hep-WD patients, which surpasses the *BrainAGE* of mild cognitive impairment and falls within the lower range of Alzheimer’s patients evaluated with the same prediction workflow [16]. Notwithstanding the significant discrepancy between predicted and chronological ages in the hep-WD group, and the descriptively higher *BrainAGE* than in controls (~ 0.5 years), the comparison of *BrainAGE* between hep-WD patients and controls did not reach statistical significance. This may be attributable to the constrained statistical power resulting from the small sample size in the hep-WD group. Additionally, *BrainAGE* is influenced not solely by disease but also by lifestyle factors, including obesity, physical activity, alcohol and nicotine consumption [42–44], which may have contributed to the large inter-individual variability and limited selectivity. In consideration of the findings of Viveiros et al. who identified subclinical volume loss in the thalamus, putamen, amygdala, and hippocampus in hep-WD patients [7], we recommend further research on neurodegeneration in hep-WD. In contrast with the prevailing phenotype classification based on initial symptoms [24], we adopted a more conservative approach and also included patients in the neuro-WD group if significant neurological impairment developed at a later stage. Given that a subset of hep-WD patients exhibited minimal UWDRS-N scores at the time of examination, although not statistically different from controls, the described trend of increasing *BrainAGE* may indicate an emerging shift from a hepatic to an overtly neurological phenotype. The absence of correlation between atrophy markers and age or disease duration suggests that atrophy progression in WD is not attributable to natural aging processes or an inevitable consequence of prolonged disease duration. Instead, it may be predominantly linked to copper overload [19], resulting from non-compliance or inadequate treatment management. In conclusion, our results imply that *BrainAGE* may prove a useful monitoring biomarker of WD, a hypothesis that should be tested in longitudinal studies.

We demonstrated diminished cognitive performance in the MMSE, RBMT-S, and SDMT in neuro-WD patients compared to controls, which is congruent with previous studies [3–5]. Furthermore, we identified GM atrophy, particularly in the striatum and thalamus, as a correlate of neurological symptoms, global cognitive function, mental processing speed, and verbal memory in WD. *BrainAGE* was correlated with UWDRS-N and SDMT scores, which resonates well with the observed relationship between *BrainAGE* and disease severity in other neurological disorders [17, 39, 45]. Nevertheless, clinical symptoms were more closely related to regional atrophy metrics. While neurological impairment, presumably dysarthria, may has confounded the SDMT assessment of mental processing speed, the remaining neuropsychological scores did not correlate with UWDRS-N scores. Thus, we surmise that the impact of striatal-thalamic atrophy on cognitive function extends beyond the scope of movement disorders. In line with our results, prior studies on WD demonstrated correlations between putaminal and caudate atrophy and increased neurological impairment [8, 11, 18], as well as poorer performance on executive function tests demanding processing speed or memory [20]. The latter may be attributable to the caudate nucleus’s role in the frontostriatal network, which is integral to verbal working memory [46]. In light of the recognized function of the basal ganglia in motor control, motor learning, and cognitive processes [47], as well as in other neurodegenerative diseases characterized by overlapping symptoms [48], it is reasonable that striatal atrophy contributes to neurological and cognitive deficits in neuro-WD.

In contrast, the clinical implications of thalamic atrophy in WD remain inconclusive. The thalamus is essential for filtering and relaying sensory and motor signals between the body and brain, thereby contributing to movement control, perception, and cognitive processes [49]. Nevertheless, the aforementioned studies did not identify significant correlations between clinical scores and thalamus volume in WD patients [8, 11, 18, 20]. A potential explanation for this discrepancy could be the presence of region-specific atrophy in the thalamus in WD [8]. A recent study identified a correlation exclusively between the volume of the right magnocellular mediodorsal thalamic nucleus and UWDRS-N scores [37]. Consistent with this observation, the thalamic atrophy in our sample was predominantly confined to the mediodorsal to anterior region. These are connected to the limbic system, a vital component for emotion regulation and memory, and the mesencephalon and brainstem, which are crucial for vegetative functions, ocular movements, and balance [50, 51]. Additionally, reduced thalamic volumes, particularly in anterior and medial nuclei, have been linked to diminished cognitive performance in memory, executive function, directed attention, and information processing speed in healthy subjects [52]. Taken together, rather than the entire thalamus, it may be specific thalamic subregions that are implicated in WD-related neurological and cognitive impairment.

The present findings imply that GM atrophy is a valuable and clinically relevant neuroimaging biomarker in WD. The borderline *BrainAGE* scores of hep-WD patients may pose a challenge to the commonly employed practice of using them as controls. We therefore propose that regular cranial MRIs with standardized assessment of biomarkers may prove beneficial for monitoring the disease course and treatment response in WD, regardless of the initial phenotype. Our study has demonstrated the great potential of automated workflows for atrophy estimation and brain age prediction, as they provided an accurate individual-level quantification of age- and sex-specific GM changes in WD patients and concurrently allowed straightforward interpretation through color-coded overlays or the aggregation of complex aging patterns into a single value. The use of such assistive tools may significantly enhance the feasibility of longitudinal comparisons in clinical contexts, a hypothesis that merits validation in future studies of WD.

Our study had limitations. Analyses were limited in statistical power, although our sample size was relatively large given the inherent challenges in prospectively collecting data from patients with rare diseases. The observed gender ratio was approximately two-thirds female, but despite atrophy being more prevalent in men with WD [10, 41], we still identified profound atrophy. As severe neurological impairment interfered with study assessments and, thus, precluded inclusion or caused early discontinuation, median UWDRS-N scores of neuro-WD patients were relatively low. Hence, *BrainAGE* in more severe or inadequately treated neuro-WD may be even higher than we observed. We mitigated the limitation of constrained generalizability of normative data by including healthy controls. However, the implementation of normative models in neurodegenerative diseases remains challenging due to the variability in datasets and potential image registration issues. Moreover, comparisons of *BrainAGE*s between studies should be interpreted with caution, when they are based on different prediction models. Finally, considering the correlative nature of the evidence concerning the relationship between atrophy markers and clinical scores, causal inferences cannot be drawn.

## Conclusions

We demonstrated that neuro-WD leads to a substantial increase in predicted brain age and striatal-thalamic atrophy patterns that are related to neurological and cognitive symptoms in WD. This study underscores the clinical relevance of GM atrophy in WD and the great potential of automated workflows to facilitate the monitoring of WD-related brain volume changes.

## Supplementary Information

Additional file 1.

## Data Availability

The datasets generated and analysed during the current study are not publicly available due to privacy reasons, but anonymized data are available from the corresponding author upon reasonable request.

## Abbreviations

ANOVA: Analysis of variance
BI: Bicaudate index
*BrainAGE*: Brain Age Gap Estimate
CAT12: Computational Anatomy Toolbox 12
CW-INT: Colour-Word Interference Test - interference trial
eNKI: Enhanced Nathan Kline Institute-Rockland Sample
GM: Grey matter
Hep-WD: Wilson disease with a hepatic phenotype
ICC: Intraclass correlation coefficients
MMSE: Mini-Mental State Examination
MRI: Magnetic resonance imaging
Neuro-WD: Wilson disease with a neurological phenotype
RBMT-S: Rivermead Behavioural Memory Test - story subtest
SDMT: Symbol Digit Modalities Test
SPM12: Statistical Parametric Mapping 12
TVW: Third ventricle width
UWDRS-N: Unified Wilson’s Disease Rating Scale neurological subscale
*veganbagel*: Volumetric estimation of gross atrophy and brain age longitudinally
WD: Wilson disease
