# Supplementary material for: Atrophy related neuroimaging biomarkers for neurological and cognitive function in Wilson disease
Source: Neurol Res Pract. 2025 Jul 1;7(1):47. doi: 10.1186/s42466-025-00401-3 (PMC12217823; doi:10.1186/s42466-025-00401-3)
Supplement: Supplementary file 1 — Additional file 1. [file 42466_2025_401_MOESM1_ESM.docx]

**Additional file 1**

**Magnetic resonance imaging acquisition**

In this study, three-dimensional T_1_-weighted magnetization-prepared rapid gradient-echo (MPRAGE) images were obtained using a 64-channel head coil. Acquisition parameters were adapted according to the acquisition protocol of the Lifespan Human Connectome Project in Aging [[1](#_ENREF_1)]: repetition time = 2,500 milliseconds (ms), echo time = 2.22 ms, inversion time = 1,000 ms, flip angle = 8°, field of view = 240*256 millimetres (mm), voxel size = 0.8 mm^3^. Foam padding was used to reduce head motion.

**Details on common radiological measurements**

As previously recommended, we used the slice where it was most visible to draw a guiding line through the long axis of the third ventricle along the interhemispheric fissure and then measured third ventricle width (TVW) in mm perpendicular to the midpoint [[2](#_ENREF_2), [3](#_ENREF_3)]. To obtain the bicaudate index (BI), measurements of the minimum distance between caudate nuclei’s heads and the transverse diameter of brain tissue at caudate level were taken in mm in the slice where the frontal horns were most clearly visible while the septum was the thinnest [[4](#_ENREF_4), [5](#_ENREF_5)].

**Clinical correlations of common radiological measurements**

Unified Wilson’s Disease Rating Scale neurological subscale (UWDRS-N) scores were moderately to strongly correlated with TVW (*r_s_* = .48, *p* = .010) and BI (*r_s_* = .53, *p* = .005). Given the significant correlation between all analysed atrophy markers and the UWDRS-N scores, generative additive models were used to determine whether the novel neuroimaging biomarkers (Brain Age Gap Estimate [*BrainAGE*], striatal-thalamic atrophy scores) explain additional variance in neurological symptoms beyond that explained by common radiological measurements (TVW, BI). The model fit was evaluated using the Akaike information criterion (AIC) and compared via a chi-square test. While the model including TVW and BI explained 81.4% of the deviance (AIC = 187.34) in UWDRS-N scores, adding *BrainAGE* and striatal-thalamic atrophy scores resulted in a significantly increased explained deviance of 87.9% (AIC = 180.36; p = .030). This finding suggests that *BrainAGE* and *veganbagel*-derived striatal-thalamic atrophy capture additional relevant variance in neurological symptoms compared to common radiological measurements and, thus, add value in characterizing patients with WD.

While the BI did not correlate with cognitive scores, a negative correlation was identified between TVW and MMSE (*r_s_* = -.51, *p* = .008) and SDMT scores (*r_s_* = -.49, *p* = .010).

**References**

1. Harms, M. P., Somerville, L. H., Ances, B. M., Andersson, J., Barch, D. M., Bastiani, M., Bookheimer, S. Y., Brown, T. B., Buckner, R. L., Burgess, G. C., Coalson, T. S., Chappell, M. A., Dapretto, M., & Yacoub, E. (2018). Extending the Human Connectome Project across ages: Imaging protocols for the Lifespan Development and Aging projects. *Neuroimage*, *183*, 972-984. <https://doi.org/10.1016/j.neuroimage.2018.09.060>

2. Dusek, P., Smolinski, L., Redzia-Ogrodnik, B., Golebiowski, M., Skowronska, M., Poujois, A., Laurencin, C., Jastrzebska-Kurkowska, I., Litwin, T., & Członkowska, A. (2020). Semiquantitative scale for assessing brain MRI abnormalities in Wilson disease: A validation study. *Movement Disorders*, *35*(6), 994-1001. <https://doi.org/10.1002/mds.28018>

3. Benedict, R. H. B., Weinstock-Guttman, B., Fishman, I., Sharma, J., Tjoa, C. W., & Bakshi, R. (2004). Prediction of neuropsychological impairment in multiple sclerosis: Comparison of conventional magnetic resonance imaging measures of atrophy and lesion burden. *Archives of Neurology*, *61*(2), 226-230. <https://doi.org/10.1001/archneur.61.2.226>

4. Siger, M., Wydra, J., Wildner, P., Podyma, M., Puzio, T., Matera, K., Stasiolek, M., & Swiderek-Matysiak, M. (2024). Differences in brain atrophy pattern between people with Multiple Sclerosis and systemic diseases with central nervous system involvement based on two-dimensional linear measures. *Journal of Clinical Medicine*, *13*(2), 333. <https://doi.org/10.3390/jcm13020333>

5. Wang, Y., Xuan, H., Zhao, T., Li, X., Li, S., & Hu, W. (2023). A study of linear measurement and clinical correlation of brain atrophy in Wilson's disease. *Frontiers in Human Neuroscience*, *17*, 1142082. <https://doi.org/10.3389/fnhum.2023.1142082>
